# Nephrometry scores and perioperative outcomes following robotic partial nephrectomy

**DOI:** 10.1590/S1677-5538.IBJU.2016.0571

**Published:** 2017

**Authors:** Renato B. Corradi, Emily A. Vertosick, Daniel P. Nguyen, Antoni Vilaseca, Daniel D. Sjoberg, Nicole Benfante, Lucas N. Nogueira, Massimiliano Spaliviero, Karim A. Touijer, Paul Russo, Jonathan A. Coleman

**Affiliations:** 1 Department of Surgery, Urology Service, Memorial Sloan Kettering Cancer Center, New York, USA;; 2 Department of Epidemiology and Biostatistics, Memorial Sloan Kettering Cancer Center, New York, USA;; 3 Departamento de Cirurgia, Serviço de Urologia, Hospital das Clínicas da UFMG, Belo Horizonte, MG, Brasil

**Keywords:** Kidney Neoplasms, Nephrectomy, Robotics

## Abstract

**Objectives:**

Based on imaging features, nephrometry scoring systems have been conceived to create a standardized and reproducible way to characterize renal tumor anatomy. However, less is known about which of these individual measures are important with regard to clinically relevant perioperative outcomes such as ischemia time (IT), estimated blood loss (EBL), length of hospital stay (LOS), and change in estimated glomerular filtration rate (eGFR) after robotic partial nephrectomy (PN). We aimed to assess the utility of the RENAL and PADUA scores, their subscales, and C-index for predicting these outcomes.

**Materials and Methods:**

We analyzed imaging studies from 283 patients who underwent robotic PN between 2008 and 2014 to assign nephrometry scores (NS): PADUA, RENAL and C-index. Univariate linear regression was used to assess whether the NS or any of their subscales were associated with EBL or IT. Multivariable linear regression and linear regression models were created to assess LOS and eGFR.

**Results:**

The three NS were significantly associated with EBL, IT, LOS, and eGFR at 12 months after surgery. All subscales with the exception of anterior/posterior were significantly associated with EBL and IT. Collecting system, renal rim location, renal sinus, exophytic/endophytic, and nearness to collecting system were significant predictors for LOS. Only renal rim location, renal sinus invasion and polar location were significantly associated with eGFR at 12 months.

**Conclusions:**

Tumor size and depth are important characteristics for predicting robotic PN outcomes and thus could be used individually as a simplified way to report tumors features for research and patient counseling purposes.

## INTRODUCTION

Partial nephrectomy (PN) is the preferred technique in the treatment of small (<4cm) and mid-size (<7cm) kidney masses and can entail varying degrees of technical challenges based on anatomic features of the tumor (1, 2). The growing interest in PN has highlighted the need for a standardized method for characterizing renal masses that provides clinically meaningful information when different approaches and techniques for PN are compared (3, 4).

In this context, different nephrometry scores (NS), principally based on renal imaging, have been proposed (5–7). Their main objective is to provide a reproducible way of characterizing anatomy and classifying renal masses with emphasis on the most surgically-relevant features. However, subscale metrics have not been validated individually and little is known about which of them are truly related to complications and outcomes after PN. Although some studies have investigated the impact of the individual components of NS on complications or other outcomes, their results should be taken cautiously because of small series (8, 9) and because they only compared subscales within the same NS (10–12). Furthermore, very few of them focused on robotic procedures (13, 14). This is important because trend analyses show that robotic PN is likely to become the most frequently performed operation for renal masses (15).

Here we assessed the utility of the RENAL and PADUA scores, their subscales, and C-index for predicting perioperative parameters and postoperative outcomes from a single institution series of robotic PN. We hypothesized that some subscales hold more value than others for predicting some of these outcomes, and that in daily practice the use of the most relevant subscales is as effective as the use of one of the complexity scores.

## MATERIALS AND METHODS

### Patients

After obtaining institutional review board approval, we identified 317 patients who underwent robotic PN between May 2008 and August 2014 at Memorial Sloan Kettering Cancer Center (MSKCC). Patients with benign histology (n=16) and whose imaging exams were not available (n=18) were excluded, leaving 283 patients with malignant tumors for final analysis. Baseline characteristics were extracted from a prospectively maintained database and included patient age, gender, American Society of Anesthesiologists (ASA) score, and race.

### Surgical Procedures

All procedures were performed by surgeons with over five years of experience in minimally invasive and robotic surgery including PN. Normothermic ischemia was utilized during sharp excisional resection and surgical repair was conducted by renorrhaphy with absorbable sutures over nitrocellulose and thrombin-based procoagulant materials. Cases were performed trans or retroperitoneally according to surgeon preference and tumor location. Early unclamping and enucleo-resection techniques were not utilized. Postoperative care was managed under a standardized clinical care pathway overseen by care providers independent from the surgical team (16). Surgical complications reported within 30 days were obtained prospectively using a standardized grading system (17).

### Outcome measures

#### Pathologic data

Kidney tumor specimens were evaluated according to standard pathology protocol. Pathologic data included tumor size and tumor stage according to the 2009 AJCC TNM classification (18).

### Postsurgical complications

Postsurgical complications within 30 days were collected prospectively and graded using the modified Clavien classification system (19). Regular correspondence with patients and their physicians ensured that treatment received outside of our institution was accounted for in the database.

### Perioperative outcomes

Estimated blood loss (EBL), ischemia time (IT) and length of stay (LOS) were collected from our prospectively maintained database. Data regarding IT and LOS were not available for 4 and 56 patients, respectively.

### Renal function outcomes

Baseline eGFR was calculated from creatinine readings using the Chronic Kidney Disease Epidemiology Collaboration (CKD-Epi) equation taken one month prior to surgery (20). Postoperative eGFR was calculated at 12 months or from the closest measurement between 6 and 12 months following surgery.

### Nephrometry scoring

RENAL scores were assigned as described by Kutikov and Uzzo (6). The components of this score are radius (R), exophytic/endophytic (E), proximity to collecting system or sinus (N), anterior/posterior (A), and location relative to polar lines (L). R, E, N and L are scored from 1 to 3. Subscale A is a categorical variable defined as anterior, posterior or X when a designation relative to anterior/posterior is not possible.

The PADUA nephrometry score, described by Ficarra et al. (5), is similar to the RENAL nephrometry score. Longitudinal location, renal rim, renal sinus, and urinary collecting system are scored with either 1 or 2 points; exophytic rate and tumor size are scored from 1 to 3 points. Tumor size was defined as the maximum tumor diameter as described in the original articles from Ficarra et al. and Kutikov et al. (5, 6),

C-index measurements are described by Simmons et al. (7) and were made according to the authors instructions contained in the original article where this NS is described (7).

All NS measurements were made by the same urologic surgeon and were based on the original studies described.

#### Statistical analyses

Univariate linear regression was used to assess the association between C-index, RENAL, PADUA and any of the RENAL or PADUA NS subs-cales with EBL and IT. Multivariable linear regression models adjusted for age and ASA score were used to determine whether NS is associated with LOS. Linear regression models adjusted for preoperative eGFR were used to assess the association of the NS with eGFR at 12 months after surgery. The effects of the renal NS on oncologic outcomes were not studied because of the limited number of events. All analyses were performed using Stata 12 (StataCorp, College Station, TX).

## RESULTS

Patient and tumor characteristics are described in Table-1. Renal nephrometry subscale scores are reported in Table-2.

**Table 1:** Patient and tumor characteristics, N = 283. Data are reported as frequency (%) or median (IQR).

Female		93 (33%)
Age		60 (51, 67)
**ASA score**		
	1	17 (6%)
	2	101 (36%)
	3	160 (57%)
	4	5 (2%)
**Pathologic T stage**		
	T0	4 (1%)
	T1	240 (85%)
	T2	8 (3%)
	T3	31 (11%)
**Pathologic N stage (N=277)**		
	N0	138 (50%)
	N1	1 (<1%)
	N2	4 (1%)
	NX	134 (48%)
**Pathologic M stage (N=231)**		
	M0	212 (92%)
	M1	6 (3%)
	MX	13 (5%)
Positive margins (N=279)		22 (8%)
Tumor size (cm) (N=280)		2.9 (2.0, 4.3)
Grade 3+ complications within 30 days		8 (3%)

**Table 2:** RENAL and PADUA subscale scores, N=283. Data are reported as frequency (%).

Collecting sinus (PADUA)		
	Absent	64 (23%)
	Dislocated/infiltrated	219 (77%)
**Renal rim (PADUA)**		
	Lateral	194 (69%)
	Medial	89 (31%)
**Renal sinus (PADUA)**		
	Absent	184 (65%)
	Renal sinus location	99 (35%)
**Sinus line (PADUA)**		
	Entirely above/below or <50% crossing	162 (57%)
	sinus lines	
	Entirely between / ≥50% crossing	121 (43%)
**Nearness to collecting sinus (RENAL)**		
	≥7mm	45 (16%)
	4 mm-7 mm	35 (12%)
	≤4 mm	203 (72%)
**Anterior/Posterior (RENAL)**		
	Anterior	114 (40%)
	Posterior	114 (40%)
	Neither	55 (19%)
**Location relative to polar line (RENAL)**		
	Entirely above/below	143 (51%)
	Crosses polar lines <50%	46 (16%)
	Entirely between / ≥50% crossing	94 (33%)
**Exophytic / endophytic (PADUA/RENAL)**		
	≥50% exophytic	108 (38%)
	<50% endophytic	96 (34%)
	Entirely endophytic	79 (28%)
**Size (PADUA/RENAL)**		
	≤4cm	183 (65%)
	4 cm - 7cm	81 (29%)
	≥7cm	19 (7%)

C-index, RENAL and PADUA scores were all significantly associated with EBL, IT, LOS, and eGFR at 12 months after surgery. The effect of a one-unit increase in RENAL score and a one-unit increase in PADUA score were similar for all outcomes.

With regard to subscales, tumor size, a component of both the PADUA and RENAL scores, was associated with perioperative outcomes: larger tumors resulted in significantly increased EBL and IT (Table-3). With the exception of the anterior/posterior scale, all other scales describing tumor location were significantly associated with EBL and IT, with tumors in complex locations or centrally located having increased EBL and longer IT (Table-3).

**Table 3:** Univariate linear regression models for the association between C-index, RENAL and PADUA total scores and subscales and peri-operative outcomes: estimated blood loss (EBL) in mL (N = 283) and ischemia time (IT) in minutes, (N=279).

		EBL (mL)			Ischemia time (min.)		
		β	95% CI	p value	β	95% CI	p value
C-Index (per 1 unit increase) (N=282)		-13	-24, −2	0.020	-2.4	-3.0, −1.9	<0.0001
RENAL (per 1 unit increase)		28	16, 39	<0.0001	2.8	2.2, 3.4	<0.0001
PADUA (per 1 unit increase)		29	18, 39	<0.0001	2.7	2.1, 3.2	<0.0001
**Collecting system**							
	Not involved	Ref.	–	0.013	Ref.	–	<0.0001
	Dislocated/infiltrated	70	15, 125		8.8	5.8, 11.8	
**Renal rim**							
	Lateral	Ref.	–	0.0002	Ref.	–	<0.0001
	Medial	93	44, 141		6.6	3.9, 9.4	
**Renal sinus**							
	Not involved	Ref.	–	<0.0001	Ref.	–	<0.0001
	Involved	127	81, 173		10.4	7.9, 12.9	
**Longitudinal (polar) location**							
	Superior/inferior	Ref.	–	0.006	Ref.	–	<0.0001
	Middle	64.5	19, 111		7.0	4.4, 9.5	
**Exophytic/endophytic**							
	≥50% exophytic	Ref.	–	0.030	Ref.	–	0.001
	1% – 50% exophytic	68	14, 122		5.0	2.0, 8.1	
	Entirely endophytic	58	1, 115		5.5	2.3, 8.7	
**Tumor radius**							
	≤4cm	Ref.	–	<0.0001	Ref.	–	<0.0001
	>4 cm and <7cm	80	29, 130		8.2	5.6, 10.9	
	≥7cm	168	77, 259		16.4	11.6, 21.2	
**Nearness to collecting system**							
	≥7mm	Ref.	–	0.008	Ref.	–	<0.0001
	<7mm and >4mm	-1.0	-88, 85		2.8	-2.0, 7.5	
	≤4mm	81	17, 144		9.7	6.3, 13.2	
**Anterior/posterior**							
	Anterior	Ref.	–	0.4	Ref.	–	0.5
	Posterior	-26	-78, 26		1.7	-1.2, 4.7	
	Neither	15	-49, 79		1.2	-2.4, 4.9	
**Polar lines**							
	Entirely above/below	Ref.	–	0.003	Ref.	–	<0.0001
	<50% crossing	78	13, 143		6.3	2.8, 9.9	
	≥50% crossing or entirely between	82	31, 133		8.2	5.4, 11.0	

**β** = difference between patients who have that subscale score and those in the reference group

**Ref. =** reference group

Regarding LOS, collecting system, renal rim location, renal sinus, exophytic/endophytic, and nearness to collecting system were significant predictors (Table-4). Conversely, tumor size was not associated with LOS. Furthermore, only renal rim location, renal sinus invasion and polar location were significantly associated with eGFR at 12 months. It has to be noted that anterior/posterior location was not significantly associated with any peri-operative or postoperative outcomes, while renal rim location and renal sinus invasion, both from PADUA, were the only subscales associated with all four outcomes.

**Table 4:** Multivariable linear regression models for the association between C-index, RENAL and PADUA total scores subscales and postoperative outcomes. The model for length of stay (LOS) in days was adjusted for ASA score and age (N = 227).

		Length of stay			eGFR at 12 months		
		β	95% CI	p value	β	95% CI	p value
C-Index (per 1 unit increase) (N=226)		-0.1	-0.2, −0.02	0.014	0.95	0.17, 1.72	0.017
RENAL (per 1 unit increase) (N=227)		0.14	0.07, 0.22	0.0002	-1.05	-1.86, −0.23	0.012
PADUA (per 1 unit increase) (N=227)		0.14	0.07, 0.21	0.0002	-1.06	-1.83, −0.29	0.007
**Collecting system**							
	Not involved	Ref.	–	0.001	Ref.	–	0.6
	Dislocated/infiltrated	0.6	0.2, 1.0		-1.12	-5.04, 2.80	
**Renal rim**							
	Lateral	Ref.	–	0.001	Ref.	–	0.019
	Medial	0.6	0.3, 0.9		-4.24	-7.76, −0.72	
**Renal sinus**							
	Not involved	Ref.	–	0.015	Ref.	–	0.003
	Involved	0.4	0.1, 0.7		-5.17	-8.58, −1.77	
**Longitudinal (polar) location**							
	Superior/inferior	Ref.	–	0.2	Ref.	–	0.014
	Middle	0.2	-0.1, 0.5		-4.15	-7.47, −0.84	
**Exophytic/endophytic**							
	≥50% exophytic	Ref.	–	0.049	Ref.	–	0.6
	1% – 50% exophytic	0.3	-0.1, 0.7		-1.97	-5.85, 1.92	
	Entirely endophytic	0.5	0.1, 0.8		-0.23	-4.35, 3.89	
**Tumor radius**							
	≤4cm	Ref.	–	0.082	Ref.	–	0.057
	>4cm and <7cm	0.4	0.04, 0.7		-2.60	-6.27, 1.06	
	≥7cm	0.3	-0.3, 0.9		-7.26	-13.85, −0.68	
**Nearness to collecting system**							
	≥7mm	Ref.	–	0.002	Ref.	–	0.11
	<7mm and >4mm	0.1	-0.5, 0.7		1.83	-4.40, 8.07	
	≤4mm	0.7	0.2, 1.1		-2.96	-7.50, 1.57	
**Anterior/posterior**							
Anterior		Ref.	–	0.6	Ref.	–	0.8
Posterior		-0.2	-0.5, 0.2		-0.78	-4.45, 2.90	
Neither		0.01	-0.4, 0.4		-1.68	-6.25, 2.89	
**Polar lines**							
	Entirely above/below	Ref.	–	0.2	Ref.	–	0.073
	<50% crossing	0.3	-0.2, 0.7		-3.05	-7.69, 1.59	
	≥50% crossing or entirely between	0.3	-0.1, 0.6		-4.14	-7.81, −0.46	
**polar lines**							
	Did not cross or <50% crossing	Ref.	–	0.069	Ref.	–	0.045
	≥50% crossing or entirely between	0.3	-0.02, 0.62		-3.56	-7.04, −0.08	
	(centrally located tumors)						

The model for post-operative eGFR at 12 months was adjusted for pre-operative eGFR (N=279).

**β** = difference in length of stay between patients with that subscale score and patients in the reference group with similar ages and ASA scores

**Ref. =** reference group

Tumor location relative to polar lines was found to significantly increase both EBL and IT, with patients with centrally located tumors having the worst outcomes. As a sensitivity analysis, we compared patients with centrally located tumors (entirely between or crossing polar lines more than 50%) with patients whose tumors did not cross polar lines or crossed less than 50%. Patients with centrally located tumors had an increase in EBL of 60mL (95% confidence interval [CI] 12, 109, p=0.02) and increase in IT of 6.5 minutes (95% CI 3.8, 9.2, p <0.0001) compared to patients without centrally located tumors. When comparing patients with centrally located tumors to all others, we found some evidence that these patients had longer LOS (0.30 days, 95% CI-0.02, 0.62, p=0.07), although this association did not reach conventional levels of statistical significance. These patients also had eGFR at 12 months that was 3.56 points lower (95% CI-7.04, −0.08, p=0.045) compared to patients with tumors that did not cross the polar line or crossed <50%.

## DISCUSSION

Prior to the introduction of NS only limited and mainly quantitative information regarding surgically-relevant anatomical features of solid enhancing renal masses was available. By defining the characteristics of tumors treated with PN, integrated anatomical systems can be used to predict the risk of surgical complications and allows comparisons among different surgeons and techniques. Given the variability in techniques and possibly outcomes inherent to surgical approaches, here we offer a comprehensive analysis on the value of all previously described NS and their subscales for robotic PN.

The RENAL and PADUA NS contain subs-cales related to tumor size and location within the renal parenchyma, as well as relative to portions of the collecting system or the renal sinus. The C—index is calculated based on the position of the tumor relative to the center of the kidney and is adjusted by tumor size. Any renal mass with a C—index <1 has some part superimposed on the renal center and a C-index of 1 equates to a tumor with its edge touching the middle of the kidney. Simmons and coworkers from the Cleveland Clinic (7) showed that a C-index <2 was associated with longer IT, higher EBL and rates of intra- and postoperative complications after laparoscopic PN. Here, concordant findings were demonstrated for robotic PN, as a one-unit increase in C-index was associated with lower EBL and shorter IT.

In the current analysis, PADUA and RENAL were associated with all outcomes studied, which is in line with previous non-robotic analyses. In a large retrospective multicenter study, Ficarra et al. showed that PADUA stratification was an independent predictor of IT longer than 20 minutes and overall complications within 3 months (12).

Smaller series also demonstrated associations between RENAL, PADUA and C-index with warm IT (4) and change in renal function (8, 21).

However, a main issue remains as to whether, for robotic PN, there is significant added value of using a composite index scoring system over the simple use of individual measures used to calculate the index. In our analysis that included a large series of robotic PN, all subscales from the PADUA and RENAL scores, except for anterior/posterior, were independently associated with two important surrogates of challenging tumor resections (1, 22), EBL and IT. Tumor size ≥7cm had the biggest impact on IT and EBL, being associated with IT on average 16 minutes and a mean EBL 168mL higher than for tumor size ≤4cm. Concordant findings were seen in a preliminary study from our institution including 90 open, laparoscopic and robotic PN procedures in which location relative to polar line, sinus involvement, tumor size, collecting system involvement, and C-index score were all associated with increased IT (9). Along these lines, Tsivian et al. reported that tumor size, endophytic growth and both central and hilar mass location were associated with increased IT (23). It has to be stressed that other parameters such as the amount of perinephric fat can bring additional difficulty to the surgical procedure (24) and affect perioperative outcomes. Along the same line, the volume of preserved parenchyma has shown prognostic value for postoperative renal function (25). Our study aimed at evaluating subscales of NS and therefore we did not evaluate these other factors.

LOS is an important outcome when evaluating surgical procedures and an accepted surrogate for immediate post-operative convalescence features including complications (1). Moreover, shortening of LOS is one of the assumed benefits of the robotic approach. Here LOS was associated with features that evaluate tumor depth of invasion, demonstrating roughly changes per each of the subscales unit increase. All cases analyzed were performed by the same operative approach (robotic PN) using a common care pathway: the LOS outcome is likely a surrogate for immediate postoperative convalescence features including complications (1). These data are consistent with those from Simhan et al. (26). Performing a multivariable analysis on 390 patients who underwent PN, the authors found that high tumor complexity using the RENAL US was associated with the occurrence of major complications. The low rate of major postoperative complications (Table-1) in our cohort precluded comparative analyses between NS and subscales.

Renal function after PN depends on several factors, mainly pre-operative eGFR, IT and the volume of preserved kidney parenchyma (1, 25, 27, 28) It seems intuitive that the more complex the renal tumors are, the longer the vessels need to be clamped to achieve safe excision and reconstruction (29). Using multivariable analysis we showed that only three PADUA subscales and nephrometry composite scores were associated with functional loss. Moreover, although the composite indexes were associated with all outcomes studied, the magnitude of difference per unit change in index score was less than for renal rim, sinus involvement and polar location. Thus, our data question the utility of composite index scoring compared to individual subscale for robotic PN.

The ideal method to describe renal masses should not only accurately reflect surgical complexity, but also be reproducible and objective in order to be applicable in daily practice. The reduction of subscales reported in each score might augment each method's reproducibility and applicability.

Although our database is prospectively maintained, LOS missing for 56 patients due to loss of electronic data. Another limitation of our study is the retrospective nature of the analysis, which makes our results vulnerable to caveats in patient selection and other unrecognized confounding factors. However, NS scoring was done prospectively as we evaluated all previously described NS. Another strength of the study was the use of a large series of robotic PN, thus limiting variabilities related to technique compared to previous studies.

In our analysis, we show that the relevant NS features are those that describe tumor depth of invasion and size. It is interesting that tumor size was a significant predictor despite the fact that median tumor size in this study was 2.9cm. Intuitively, this would be expected for larger tumors only. This suggests potential for solely using subscales as a simplified way to stratify kidney tumors for case mix adjustments in programs of quality assurance, for example, when comparing the outcomes of different surgeons and techniques. Furthermore, individual subscale metrics can be a simplified way for the practicing urologist in his/her clinical practice to counsel patients about surgical risks.
